# Automated Hippocampal Subfield Measures as Predictors of Conversion from Mild Cognitive Impairment to Alzheimer’s Disease in Two Independent Cohorts

**DOI:** 10.1007/s10548-014-0415-1

**Published:** 2014-11-05

**Authors:** Wasim Khan, Eric Westman, Nigel Jones, Lars-Olof Wahlund, Patrizia Mecocci, Bruno Vellas, Magda Tsolaki, Iwona Kłoszewska, Hilkka Soininen, Christian Spenger, Simon Lovestone, J-Sebastian Muehlboeck, Andrew Simmons, for the AddNeuroMed consortium and for the Alzheimer’s Disease Neuroimaging Initiative

**Affiliations:** 1 Department of Neuroimaging, Institute of Psychiatry, King’s College London, De Crespigny Park, London, SE5 8AF UK; 2 Department of Neurobiology, Care Sciences and Society, Karolinska Institutet, Stockholm, Sweden; 3 Institute of Gerontology and Geriatrics, University of Perugia, Perugia, Italy; 4 INSERM U 558, University of Toulouse, Toulouse, France; 5 Aristotle University of Thessaloniki, Thessaloniki, Greece; 6 Medical University of Lodz, Lodz, Poland; 7 Department of Neurology, University of Eastern Finland and Kuopio University Hospital, Kuopio, Finland; 8 Department of Clinical Science, Intervention and Technology, Karolinska Institutet, Stockholm, Sweden; 9 NIHR Biomedical Research Centre for Mental Health, London, UK

**Keywords:** Alzheimer’s disease, MCI conversion, MRI, Freesurfer, OPLS

## Abstract

Previous studies have shown that hippocampal subfields may be differentially affected by Alzheimer’s disease (AD). This study used an automated analysis technique and two large cohorts to (1) investigate patterns of subfield volume loss in mild cognitive impairment (MCI) and AD, (2) determine the pattern of subfield volume loss due to age, gender, education, *APOE* ε4 genotype, and neuropsychological test scores, (3) compare combined subfield volumes to hippocampal volume alone at discriminating between AD and healthy controls (HC), and predicting future MCI conversion to AD at 12 months. 1,069 subjects were selected from the AddNeuroMed and Alzheimer’s disease neuroimaging initiative (ADNI) cohorts. Freesurfer was used for automated segmentation of the hippocampus and hippocampal subfields. Orthogonal partial least squares to latent structures (OPLS) was used to train models on AD and HC subjects using one cohort for training and the other for testing and the combined cohort was used to predict MCI conversion. MANCOVA and linear regression analyses showed multiple subfield volumes including Cornu Ammonis 1 (CA1), subiculum and presubiculum were atrophied in AD and MCI and were related to age, gender, education, *APOE* ε4 genotype, and neuropsychological test scores. For classifying AD from HC, combined subfield volumes achieved comparable classification accuracy (81.7 %) to total hippocampal (80.7 %), subiculum (81.2 %) and presubiculum (80.6 %) volume. For predicting MCI conversion to AD combined subfield volumes and presubiculum volume were more accurate (81.1 %) than total hippocampal volume. (76.7 %).

## Introduction

In recent years, research efforts in Alzheimer’s disease (AD) have focused upon the discovery of clinically meaningful and non-invasive biomarkers that can reliably monitor disease progression and predict future conversion to the disease. Several groups including our own have proposed the use of magnetic resonance imaging (MRI) based tools to aid in the diagnosis of AD (Desikan et al. 2009; Liu et al. 2009) and predict future conversion from the prodromal stage of disease often referred to as mild cognitive impairment (MCI) (McEvoy et al. 2009; Westman et al. 2011a).

Hippocampal atrophy has been frequently observed in AD (Jack et al. 1992; Fox et al. 1996) and has been demonstrated in MCI subjects (Devanand et al. 2007), with increased risk of future conversion to AD in subjects with smaller hippocampal volumes (Apostolova et al. 2006; Csernansky et al. 2005). Hippocampal volumetry has been a useful marker of AD pathology but seems to be insufficiently sensitive for distinguishing between MCI subjects bearing a high risk of AD conversion from those who remain clinically stable (Mueller et al. 2010). Post-mortem studies have also demonstrated that hippocampal atrophy in AD is non-uniform, with Cornu Ammonis 1 (CA1) and subicular atrophy reported in early AD (Braak and Braak 1991; West et al. 2004).

So far, only a few studies have attempted to measure regional atrophic changes within the hippocampus using manual delineation and 3D surface mapping (Mueller and Weiner 2009; Apostolova et al. 2010; Costafreda et al. 2011). Manual delineation of the subfield boundaries is both a time consuming and labour intensive process which limits its widespread applicability in practice. However, recent developments in image acquisition have made it possible to segment the hippocampus into its subfields in a fully automated fashion and this method has now been validated using ultra high resolution MRI (Van Leemput et al. 2009). A recent small study applied this technique to 15 MCI subjects using conventional 3D T_1_ weighted volume imaging and demonstrated that segmenting subfields increased sensitivity in diagnosing MCI (Hanseeuw et al. 2011). The current study uses an extensive dataset created by combining two large cohorts from the AddNeuroMed and the Alzheimer Disease Neuroimaging Initiative (ADNI) studies to build on and extend this earlier work.

In this study we aimed to (1) investigate the differences in hippocampal subfields between subject groups at baseline in a cohort of 1,069 subjects, (2) determine patterns of subfield volume loss in relation to age, gender, education, *APOE* ε4 genotype, and neuropsychological tests from mini mental state exam (MMSE) and Alzheimer disease Assessment Score-1 (ADAS-1) scores, and (3) compare combined subfield volumes using orthogonal partial least squares (OPLS) multivariate analysis to hippocampal volume alone for discriminating between AD and healthy control (HC) subjects and predicting future conversion from MCI to AD at 12 months.

## Materials and Methods

### Study Data and Inclusion and Diagnostic Criteria

The data used in this study were derived from two large multicentre cohorts, the AddNeuroMed and ADNI cohorts. The AddNeuroMed study is an integrated project funded by the European Union Sixth Framework Program and aims to establish and validate novel biomarkers of disease and treatment based upon in vitro and in vivo human and animal models of AD. Data was collected from six participating sites across Europe: University of Kuopio, Finland; University of Perugia, Italy; Aristotle University of Thessaloniki, Greece; King’s College London, United Kingdom; University of Lodz, Poland; and University of Toulouse, France (Lovestone et al. 2009; Simmons et al. 2009, 2011).

Data from the ADNI study was downloaded from the ADNI at the LONI website (www.loni.ucla.edu/ADNI, PI Michael M. Weiner). The initiative was launched in 2003 by the National Institute on Ageing, the National Institute of Biomedical Imaging and Bioengineering, the Food and Drug Administration, private pharmaceutical companies and non-profit organisations, as a 5 years public–private partnership. The primary goal of ADNI has been to test whether MRI, positron emission tomography (PET), and other biological markers are useful in clinical trials of MCI and early AD. Subjects aged 55–90 from over 50 sites across the U.S and Canada participated in the research, and imaging, clinical, and biological samples were collected at multiple time points (Jack et al. 2008). A detailed description of the inclusion criteria for the study can be found on its webpage (http://www.adni-info.org/scientists/aboutADNI.aspx#).

A total of 1,069 subjects were included in this study (AD = 291, MCI = 447, HC = 331). The demographics of the cohorts are given in Table 1. Of the 447 MCI subjects in our whole cohort, 90 converted to an AD diagnosis (MCI converters) at 12 months.

**Table 1:** Demographic, clinical and neuropsychological data in AD, MCI converters, stable MCI, and control subjects

	AD (*n* = 291)	MCI converters (*n* = 90)	Stable MCI (*n* = 357)	HC (*n* = 331)	p*
Gender (male/female)	131/160^a^	54/36	216/141^b^	166/165	0.001
Age	75.4 ± 7.0	74.1 ± 6.6	75.1 ± 7.0	75.0 ± 5.7	0.439
Years of education	12.1 ± 4.7^a,b,c^	14.0 ± 4.1	14.3 ± 4.4	14.3 ± 4.4	<0.001
MMSE	22.4 ± 3.4^a,b,c^	26.5 ± 1.8^b^	27.1 ± 1.7^b^	29.1 ± 1.1	<0.001
ADAS-1	6.3 ± 1.5^a,b,c^	5.3 ± 1.3^a,b^	4.6 ± 1.4^b,c^	3.1 ± 1.3	<0.001
CDR	0.9 ± 0.4^a,b,c^	0.5^b^	0.5^b^	0	<0.001
*APOE* ε4 genotype (+ive/-ive)	183/108	57/33	171/186	93/238	<0.001

Data are represented as mean ± and standard deviation. Chi square was used for gender and *APOE* ε4 genotype comparison. ANOVA with Bonferroni post hoc test was used for age, education, and neuropsychological scores

^a^Significant compared to stable MCI

^b^Significant compared to healthy controls (HC)

^c^Significant compared to MCI converters

* *p* values corrected for multiple comparisons using the Bonferroni method

For the AddNeuroMed cohort, subjects were patients who attended local memory clinics and received a diagnosis of MCI while HC subjects were recruited from non-related members of the patient’s families, caregiver relatives, and social centres for the elderly or General Practitioner (GP) surgeries. Informed consent was obtained for all subjects and the study was approved by the ethical review boards of each participating country. The general inclusion and exclusion criteria were as follows.

### AD

(1) diagnosis established by National Institute of Neurological and Communicative Disorders and Stroke and the Alzheimer’s Disease and Related Disorders Association (NINCDS-ADRDA) and Diagnostic and Statistical Manual of Mental Disorders IV (DSM IV) criteria, (2) MMSE score ranged from 12 to 28. Subjects were excluded from the study if any psychiatric or neurological illness other than AD was present, and if subjects presented with a systemic illness or signs of organ failure.

### MCI

(1) subjects had MMSE scores between 24 and 30, (2) subjective memory complaint with preserved activities of daily living, (3) Clinical Dementia Rating (CDR) score of 0.5, (4) Geriatric depression scale score less than or equal to 5, (5) absence of dementia in accordance with NINCDS-ARDA criteria. A 12 months follow up was used to determine whether MCI subjects converted to AD (MCI converters) or remained clinically stable (stable MCI).

### HC

(1) MMSE scores between 24 and 30, (2) CDR score of 0, (3) no presence of neurological or psychiatric illness, and non-demented.

MMSE, CDR, and the Consortium to Establish a Registry for Alzheimer’s Disease (CERAD) cognitive battery were assessed for each subject. The CERAD cognitive battery was replaced with the Alzheimer’s disease assessment scale for AD subjects in AddNeuroMed. The CERAD battery employs the same 10 word recall task as the Alzheimer’s assessment scale, only the scoring is inverted. Therefore, the mean number of words not recalled in the CERAD word list task was calculated in order to obtain comparable measures of memory for all diagnostic groups. This revised cognitive parameter was named ADAS-1 corresponding to the first subtest of the Alzheimer’s disease assessment scale.

### MRI Acquisition

Standardized MRI data acquisition techniques were in place for AddNeuroMed and ADNI to ensure homogeneity across data acquisition sites. A detailed description of the ADNI data acquisition protocol can be found at www.loni.ucla.edu/ADNI/research/Cores/index.shtml. The imaging protocol included a 1.5T high resolution T1 weighted sagittal 3D MP-RAGE volumes (voxel size 1.1 × 1.1 × 1.2 mm^3^), and axial proton density with T2 weighted fast spin echo images. A comprehensive quality control procedure was carried out on all MR images according to the AddNeuroMed quality control framework (Simmons et al. 2009, 2011).

### Hippocampal Subfield Segmentation

Image analysis was carried out using the Freesurfer image analysis pipeline (version 5.1.0). These procedures have been described in detail in previous publications (Dale et al. 1999; Fischl et al. 2002; Ségonne et al. 2004; Fischl et al. 2004). Initially volumetric segmentation involved the removal of non-brain tissue using a hybrid watershed/surface deformation procedure (Ségonne et al. 2004), automated Talairach transformation, segmentation of the subcortical white matter and deep grey matter volumetric structures (Fischl et al. 2004).

**Fig. 1:**
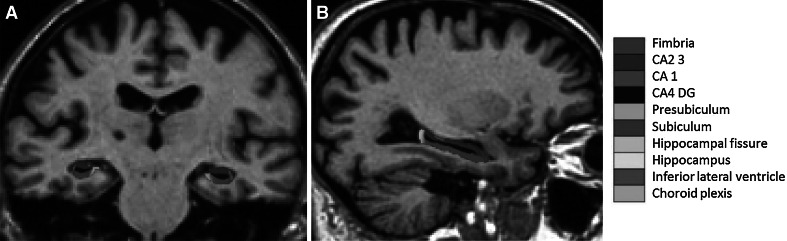
**a** Coronal and **b** sagittal views of the hippocampus

Automated segmentation of the hippocampus was performed to define anatomical subfield labels using a Bayesian modelling approach and a computational model of the areas surrounding the hippocampus. An atlas mesh had previously been built and validated from manual delineations in ultra-high resolution MRI scans of 10 individuals (Van Leemput et al. 2009). These delineations include the fimbria, presubiculum, subiculum, CA1, CA2/3, and CA4-DG subfields as well as the hippocampal fissure. Figure 1 illustrates the delineations made to define the different subfields of the hippocampus. For more details about this technique and the borders used to define the different subfields, see Van Leemput et al. (2009).

All subfield measures were normalised by the subject’s intracranial volume derived from Freesurfer using the following formula: volume_norm_ = volume_raw_ × 1,000/ICV in cm^3^ (Westman et al. 2013). This automated segmentation approach has been recently applied to a small group of MCI subjects (Hanseeuw et al. 2011).

### Statistical Analysis

Statistical analysis was conducted using PASW Statistics (Version 17. 0; SPSS inc., USA). Categorical variables were inspected using the Chi square test while continuous variables were tested using ANOVA with Bonferroni post hoc comparisons. Hippocampal subfield volumes were first analysed using MANCOVA utilising Bonferroni correction by adopting a general linear model procedure, adjusting for age, gender, education, and *APOE* ε4 genotype as covariates. Bonferroni pairwise comparisons were performed to inspect subfield volume differences between the groups.

Multiple regression analyses were conducted in R version 2.15.2 using the lm function from the R stats package and Bonferroni correction for multiple comparisons. Patterns of subfield volume loss were tested in relation to the effects of age, gender, education, *APOE* ε4 genotype, and neuropsychological test scores from MMSE and ADAS-1. In this step, all subfield measures were tested as dependent variables by disease group (AD, MCI converters, stable MCI, and HC) as a whole. Age, gender, years of education, *APOE* ε4 genotype, and neuropsychological scores from MMSE and ADAS-1 tests was treated as independent variables for identifying subfield specific effects. 10 fold cross validation was performed by fitting linear regression models to the data, excluding 1/10th of the data in each fold and using the fitted model for prediction on data that was excluded from the fold.

Hippocampal subfields were subsequently analysed using Orthogonal Partial Least Squares (OPLS) (Wiklund et al. 2008; Trygg and Wold 2002), a supervised multivariate data analysis method included in the software package SIMCA (Umetrics AB, Umea, Sweden). Al1 14 variables (left and right subfields) were used for OPLS analysis. Classification models were created for distinguishing between AD and HC subjects at baseline. The AD versus HC models were subsequently treated as classifiers to investigate how well the hippocampal subfields could predict future MCI conversion to AD at 12 months follow up. Seven-fold cross validation was used for all models. Using this approach we created 4 OPLS models; 2 for the total hippocampus and 2 for the combination of subfield volumes. The first model for each region comprised the AddNeuroMed cohort and the second model comprised the ADNI cohort. To further validate the models created the AddNeuroMed cohort was used as the training set and the ADNI cohort as a test set (and vice versa) to see how well the models could predict new and unseen data. The combined ADNI and AddNeuroMed cohort from the AD versus HC comparison was used as a classifier to investigate the reliability of predicting MCI conversion to AD at 12 months. This OPLS classification approach has been extensively validated (Bylesjo et al. 2006; Wiklund et al. 2008; Westman et al. 2011c) and applied to several biomarker discovery studies in AD (Mangialasche et al. 2010; Westman et al. 2011a, 2012; Spulber et al. 2013).

Sensitivity and specificity were calculated from the cross-validated prediction values of the OPLS models. The positive and negative likelihood ratios (LR+ = sensitivity/(100−specificity) and LR− = (100−sensitivity)/specificity)) were determined. A positive likelihood ratio between 5 and 10 or a negative likelihood ratio between 0.1 and 0.2 increases the diagnostic value in a moderate way, while a value above 10 or below 0.1 significantly increases the diagnostic value of the test.

Receiver operating characteristic (ROC) curves were calculated for the individual subfield volume models using the ROCR library (version 2.1) in R. ROC curves provide a graphical means to interpret the quality of separation and are created by plotting the true positive rate (sensitivity) versus the false positive rate (1−specificity) for various thresholds. The discriminant value of the corresponding ROC curve can be obtained by calculating the area under the curve (AUC). AUC values range from 0.5 (random discriminations no better than chance) to 1.0 (perfect discrimination). The pROC (Receiver Operating Characteristic) package (version 1.5.4) (Robin et al. 2011) in R was used to perform area under the curve (AUC) statistical comparisons between the combined subfield and total hippocampal volume models in the AD vs. HC and MCI converter vs MCI non-converter models.

## Results

### Demographics, Neuropsychological, and Global Clinical Measurements

1,069 subjects were included in the current study (AD = 291, MCI = 447, HC = 331) from the AddNeuroMed and ADNI cohorts. Results from global, clinical and cognitive assessments revealed that scores on MMSE, CDR, and ADAS-1 were poorest amongst AD and best amongst control subjects as expected (Table 1).

### Hippocampal Subfields

**Fig. 2:**
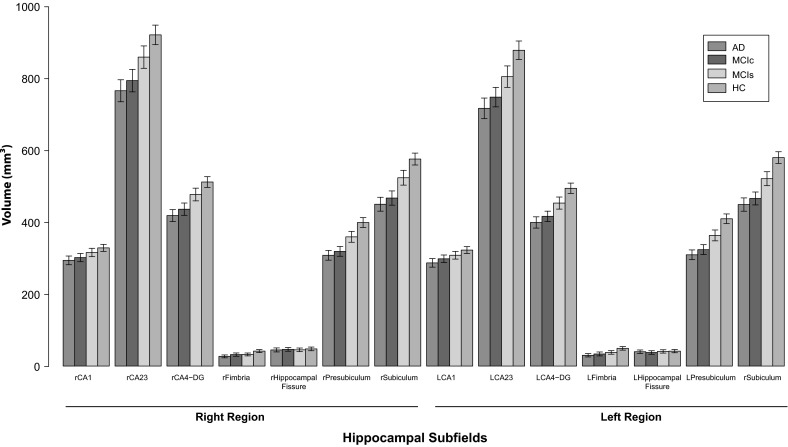
*Bar* plot of subfield volumes of AD (*n* = 291), MCI converters (*n* = 90), MCI stable (*n* = 357), and healthy control (HC) subjects (*n* = 331). *Error bars* represent SEM = SD/√n. Subfield volumes are represented in mm3. R = subfield volumes from the *right hemisphere*, L = subfield volumes from the *left hemisphere*

Hippocampal subfield volumes from the left and the right hemisphere were used to determine the pattern of subfield atrophy in AD, MCI-converter, MCI stable and HC subjects. Comparisons of the bilateral CA1, CA2-3, CA4-DG, subiculum, and presubiculum were significant across all groups (<0.0001) after correction for multiple comparisons and demonstrated similar results in pairwise comparisons (Table 2 and Fig. 2). No significant volume differences were found for the left and right hippocampal fissure between these groups.

**Table 2:** Hippocampal subfield differences in AD, MCI converters, stable MCI, and healthy control subjects

	AD (*n* = 291)	MCI converters (*n*− = 90)	Stable MCI (*n* = 357)	HC (*n* = 331)	*p*
Left presubiculum	309.2 ± 63.5^a,b^	323.7 ± 64.6^a,b^	363.2 ± 70.9^b,c^	409.5 ± 62.963.1	<0.0001
Left subiculum	449.0 ± 86.7^a,b^	465.9 ± 84.1^a,b^	520.9 ± 91.0^b,c^	579.5 ± 76.2	<0.0001
Right presubiculum	307.9 ± 64.0^a,b^	318.7 ± 63.5^a,b^	359.2 ± 70.8^b,c^	399.0 ± 64.3	<0.0001
Right subiculum	449.9 ± 90.4^a,b^	467.1 ± 92.7^a,b^	523.5 ± 96.4^b,c^	575.5 ± 77.5	<0.0001
Left CA4-DG	399.2 ± 73.5^a,b^	416.2 ± 67.6^a,b^	453.1 ± 79.0^b,c^	494.2 ± 67.7	<0.0001
Left CA2-3	716.4 ± 134.2^a,b^	747.6 ± 127.1^a,b^	804.6 ± 139.8^b,c^	877.9 ± 121.0	<0.0001
Right CA4-DG	418.3 ± 78.0^a,b^	436.1 ± 80.6^a,b^	476.9 ± 83.2^b,c^	511.6 ± 69.8	<0.0001
Right CA2-3	765.4 ± 143.4^a,b^	793.5 ± 146.4^a,b^	858.8 ± 146.0^b,c^	920.7 ± 127.7	<0.0001
Left fimbria	29.9 ± 21.5^a,b^	33.6 ± 25.6^b^	37.9 ± 23.3^b^	49.1 ± 23.1	<0.0001
Left CA1	286.8 ± 55.5^a,b^	298.2 ± 49.1^b^	308.1 ± 50.1^b^	322.4 ± 45.0	<0.0001
Right CA1	293.8 ± 57.0^a,b^	301.5 ± 53.2^b^	315.7 ± 54.2^b,c^	328.5 ± 45.8	<0.0001
Right fimbria	26.9 ± 18.8^a,b^	31.8 ± 21.5^b^	32.3 ± 18.8^b^	41.5 ± 19.9	<0.0001
Right hippocampal fissure	44.8 ± 26.2	46.2 ± 23.8	45.1 ± 23.8	47.7 ± 24.1	2.144
Left hippocampal fissure	39.8 ± 22.7	37.6 ± 22.8	40.7 ± 21.6	41.4 ± 21.1	3.276

Absolute Hippocampal subfields are presented (mm^3^). However, normalised hippocampal subfields (absolute hippocampal subfield/intracranial volume) were used in MANCOVA with Bonferroni pairwise comparisons. Age, gender, education, and *APOE* ε4 genotype were used as covariates. *p* values were corrected for multiple comparisons using the Bonferroni method

^a^Significant compared to stable MCI

^b^Significant compared to healthy controls (HC)

^c^Significant compared to MCI converters

In the left hippocampus, presubiculum (*F* = 144.5, *p* < 0.0001) and subiculum (*F* = 144.3, *p* < 0.0001) volumes were most significantly reduced in AD, MCI converter and MCI stable subjects compared to healthy controls. The same pattern of subfield atrophy was also observed in the right hippocampus for these groups for both presubiculum (*F* = 122.1, *p* < 0.0001) and subiculum (*F* = 120.0, *p* < 0.0001) relative to healthy controls. MCI-converters displayed significant subfield volume losses in the bilateral subiculum (right = *p* < 0.0001, left = *p* < 0.0001), subiculum (right = *p* < 0.0001, left = *p* < 0.0001), CA4-DG (right = *p* < 0.0001, left = *p* < 0.0001), and CA2-3 (right = *p* < 0.0001, left = *p* < 0.0001) relative to stable MCI subjects. However, no significant differences in any of the subfield volume measures were observed between AD and MCI-converter subjects.

### Relationship Between Neuropsychological Test Scores and Hippocampal Subfields

A significant positive effect for MMSE was found in relation to all hippocampal subfield volumes except bilateral hippocampal fissure, indicating that subjects with lower MMSE scores had reduced subfield volumes (Table 3). On the other hand, a significant negative effect was observed for ADAS-1 scores across all subfield volumes except the bilateral hippocampal fissure, indicating that subjects with higher ADAS-1 scores (mean number of words not recalled) had lower subfield volumes (Table 3).

**Table 3:** MMSE and ADAS-1 effect on hippocampal subfield volumes in the combined cohort

	MMSE score			ADAS-1 score		
Hippocampus	β	*t*	p*	β	*t*	p*
Left CA1	0.24	8.41	<0.001	−0.19	−6.68	<0.001
Right CA1	0.23	8.15	<0.001	−0.16	−5.47	<0.001
Left CA2-3	0.34	12.29	<0.001	−0.34	−11.93	<0.001
Right CA2-3	0.35	12.40	<0.001	−0.28	−9.67	<0.001
Left CA4-DG	0.37	13.47	<0.001	−0.36	−12.51	<0.001
Right CA4-DG	0.37	13.31	<0.001	−0.31	−10.65	<0.001
Left Fimbria	0.23	8.08	<0.001	−0.23	−8.07	<0.001
Right Fimbria	0.19	6.51	<0.001	−0.18	−6.19	<0.001
Left Presubiculum	0.43	15.51	<0.001	−0.42	−15.19	<0.001
Right Presubiculum	0.40	14.11	<0.001	−0.39	−13.60	<0.001
Left Subiculum	0.43	15.57	<0.001	−0.42	−15.06	<0.001
Right Subiculum	0.40	14.49	<0.001	−0.36	−12.67	<0.001

Age, gender, education and *APOE* ε4 genotype were introduced as covariates in these models

* p values from the regression models were corrected for multiple comparisons using the Bonferroni method

### Relationship Between Age, Education, *APOE* ε4 Genotype, and Hippocampal Subfields

A significant negative effect of age was observed in relation to all subfield volumes indicating that older subjects had lower hippocampal subfield volumes. In particular, the strongest effects of age were found in the right presubiculum (β = −0.32, *p* < 0.001), and left presubiculum areas, the right fimbria (β = −0.31, *p* < 0.001), and the right subiculum and left subiculum areas (β = −0.28, *p* < 0.001).

Linear regression models were also created to test for the effect of gender on subfield volume differences in the male (*n* = 567) and female (*n* = 502) subjects. A significant positive effect of gender was found in the right fimbria and left fimbria areas, and in the right and left CA4-DG subfield volumes (Table 4).

**Table 4:** Age and Gender effect on hippocampal subfields in the combined cohort

Hippocampus	Age^a^				Gender^b^			
	β	*t*	PRESS	*p* value*	β	*t*	PRESS	*p* value*
Left CA1	−0.1	−3.31	42.8	0.012	0.04	1.16	0.239	2.964
Right CA1	−0.09	−3.01	42.7	0.036	0.04	1.3	0.239	2.304
Left CA2-3	−0.24	−7.75	41.0	<0.001	0.09	2.93	0.237	0.036
Right CA2-3	−0.23	−7.62	40.8	<0.001	0.1	3.2	0.237	0.012
Left CA4-DG	−0.24	−7.92	40.6	<0.001	0.1	3.19	0.237	0.012
Right CA4-DG	−0.25	−8.2	40.7	<0.001	0.12	3.74	0.236	<0.001
Left Fimbria	−0.25	−8.42	39.6	<0.001	0.12	3.74	0.236	<0.001
Right Fimbria	−0.29	−9.72	40.5	<0.001	0.13	4.24	0.235	<0.001
Left Presubiculum	−0.31	−10.28	40.0	<0.001	0.09	2.71	0.238	0.084
Right Presubiculum	−0.32	−10.91	40.1	<0.001	0.09	2.89	0.237	0.048
Left Subiculum	−0.28	−9.09	38.9	<0.001	0.12	3.85	0.236	0.012
Right Subiculum	−0.28	−9.25	39.3	<0.001	0.12	3.38	0.236	0.012

^a^Gender, years of education, and APOE E4 genotype were used as covariates

^b^Subject age, years of education, and APOE E4 genotype were used as covariates

* *p values* from each regression model were corrected for multiple comparisons using the Bonferroni method

A significant negative effect of education was only found in the right CA1 (β = −0.95, *p* = 0.024).

The analysis was repeated for subjects that were carriers and non-carriers of the *APOE* ε4 allele. APOE E4 genotype was negatively related to all subfield volumes suggesting that subjects with an APOE E4 allele had smaller subfield volumes (Table 5).

**Table 5:** Years of education and APOE genotype effect on hippocampal subfields in the combined cohort

	Years of education^a^			APOE E4 genotype^b^		
	β	*t*	p*	β	*t*	p*
Left CA1	−0.59	−1.92	0.66	0.22	−7.29	0.012
Right CA1	−0.95	−3.11	0.024	0.17	−5.74	0.012
Left CA2-3	−0.004	−0.14	10.668	0.24	−7.89	<0.001
Right CA2-3	−0.01	−0.45	7.86	0.26	−8.46	<0.001
Left CA4-DG	−0.002	−0.06	11.46	0.253	−8.31	<0.001
Right CA4-DG	−0.009	−0.27	9.408	0.27	−8.8	<0.001
Left Fimbria	0.028	0.88	4.548	0.1	−3.05	0.024
Right Fimbria	0.08	2.39	0.204	0.07	−2.28	0.276
Left Presubiculum	0.03	0.81	5.004	0.24	−7.8	<0.001
Right Presubiculum	0.05	1.57	1.404	0.23	−7.28	<0.001
Left Subiculum	0.03	1.06	3.48	0.27	−8.84	<0.001
Right Subiculum	0.02	0.65	6.216	0.27	−8.66	<0.001

^a^Subject age, gender, and APOE E4 genotype were used as covariates

^b^† Subject age, gender and years of education were used as covariates

* *p values* from each regression model were corrected for multiple comparisons using the Bonferroni method

### AD and HC Classification for the Combined AddNeuroMed and ADNI Cohort

For the joint AddNeuroMed and ADNI AD versus HC model, combining the subfield volumes resulted in an accuracy of 81.7 % (sensitivity = 80.4 %, specificity = 82.8 %, AUC = 0.895) compared to 80.7 % for total hippocampal volume (sensitivity = 79.2 %, specificity = 82.8 %, AUC = 0.887) (Table 6). These result were statistically significantly different in terms of the observed AUC differences between the two models (AUC difference = 0.008, *p* = 0.001).

**Table 6:** Comparison of performance for the different cohort models in the AD vs. HC classification

	Total Hippocampus					Hippocampal subfields				
	ACC (%)	SENS (%)	SPE (%)	AUC	Q^2^ (Y)	ACC (%)	SENS (%)	SPE (%)	AUC	Q^2^ (Y)
AddNeuroMed (cv)	80.7 (74.8–85.4)	76.2 (67.2–83.3)	85.1 (77.1–90.6)	0.897	0.439	80.2 (74.3–85.0)	78.1 (69.3–84.9)	82.2 (73.9–88.3)	0.90	0.441
ADNI (cv)	81.5 (77.4–84.9)	81.2 (75.0–86.2)	81.7 (76.1–86.2)	0.884	0.404	82.0 (77.9–85.6)	81.2 (75.0–86.2)	82.6 (77.1–87.0)	0.892	0.433
Combined (cv)*	80.7 (77.4–83.6)	79.2 (74.1–83.5)	82.8 (78.3–86.5)	0.887	–	81.7 (78.4–84.5)	80.4 (75.5–84.6)	82.8 (78.3–86.5)	0.895	–
AddNeuroMed^a^	81.1 (75.3–85.8)	75.2 (66.2–82.5)	86.9 (79.2–92.0)	0.897	–	82.1 (76.4–86.7)	77.1 (68.2–84.1)	86.9 (79.2–92.0)	0.90	–
ADNI^b^	80.2 (76.1–83.8)	84.4 (78.5–88.9)	76.8 (70.8–81.8)	0.884	–	80.7 (76.6–84.3)	88.2 (82.7–92.1)	74.6 (68.5–79.8)	0.881	–

^a^AddNeuroMed dataset used as the test set and ADNI data as the training set

^b^ADNI data used as the test set and AddNeuroMed dataset as the training set

* AddNeuroMed and ADNI cohorts used as the combined cohort model, confidence intervals presented within parenthesis

*CV* cross validation, *AUC* area under the curve

**Fig. 3:**
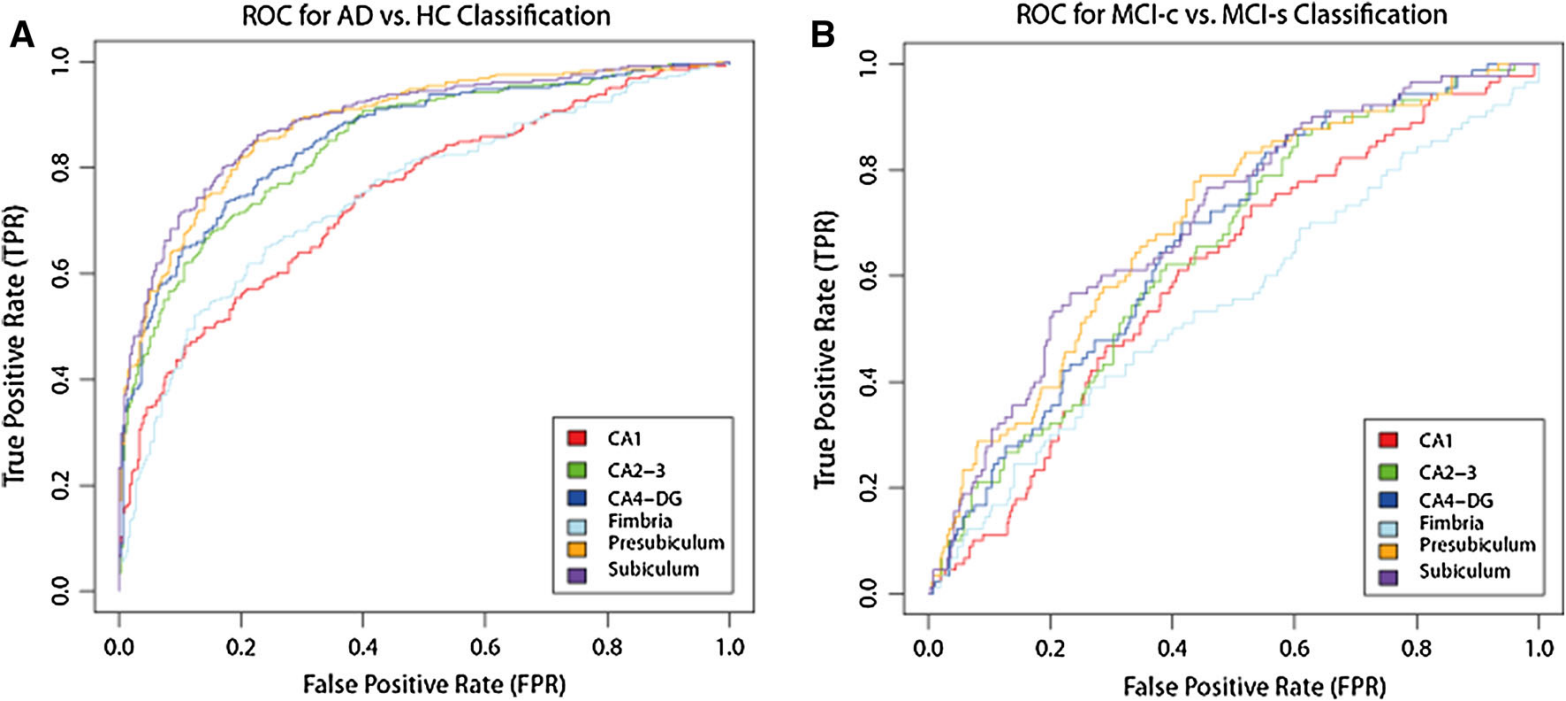
**a** ROC curve for AD versus HC classification using individual subfield measures, **b** ROC curve for *MCI-c* and *MCI-*s classification using individual subfield measures. The curve is calculated with a 95 % probability assurance. *ROC* receiver operating characteristic, *AD* Alzheimer’s disease, *HC* healthy control, *MCI-c* MCI-converter, *MCI-s* stable MCI

Combining subfield volumes resulted in similar classification accuracy to the subiculum (accuracy = 81.2 %, sensitivity = 83.5 %, specificity = 79.2 %, AUC = 0.887) and presubiculum alone (accuracy = 80.6 %, sensitivity = 83.2 %, specificity = 78.3 %, AUC = 0.882), but higher accuracies than the other individual subfield volume measures (Table 7). Figure 3 illustrates ROC curves for the corresponding individual subfield volumes for distinguishing AD and HC subjects.

**Table 7:** Comparison of performance for OPLS AD vs. HC classification models

	ACC (%)	SEN (%)	SPE (%)	AUC	PPV (%)	NPV (%)	LR+	LR−
CA1	66.0 (62.2–69.6)	68.0 (62.5–73.1)	65.9 (60.6–70.8)	0.749	63.7	70.1	1.99 (1.68–2.36)	0.49 (0.40–0.58)
CA2-3	75.4 (71.9–78.6)	77.0 (71.8–81.4)	74.0 (69.0–78.5)	0.843	72.3	78.5	2.96 (2.44–3.59)	0.31 (0.25–0.39)
CA4-DG	76.9 (73.4–80.0)	79.7 (74.7–83.9)	74.3 (69.4–78.7)	0.853	73.2	80.7	3.11 (2.56–3.76)	0.27 (0.22–0.35)
Fimbria	68.2 (64.4–71.7)	69.8 (64.3–74.8)	66.8 (61.5–71.6)	0.745	64.9	71.5	2.10 (1.77–2.49)	0.45 (0.37–0.55)
Presubiculum	80.6 (74.9–83.4)	83.2 (78.4–87.0)	78.3 (73.5–82.4)	0.882	77.1	84.1	3.08 (3.10–4.72)	0.22 (0.17–0.28)
Subiculum	81.2 (77.9–84.1)	83.5 (78.8–87.3)	79.2 (74.5–83.2)	0.887	77.9	84.5	4.01 (3.23–4.97)	0.21 (0.16–0.27)

Confidence intervals are presented within parenthesis. *AUC* area under the curve, *PPV* positive predictive value, *NPV* negative predictive value, *LR+* positive likelihood ratio, *LR−* negative likelihood ratio

### Model Validation for AD and HC Classification

Seven fold cross validation was used to determine the robustness of all the models. In this study models were validated using an external test set. The ADNI model was used as a training set and predictions were made using the AddNeuroMed cohort as the external test set and vice versa. The results are similar to those obtained by cross validation (Table 6). For the combination of hippocampal subfields, using the AddNeuroMed cohort as the test set and the ADNI cohort as the training set resulted in a similar classification accuracy, 82.1 % (sensitivity = 77.1 %, specificity = 86.9 %, AUC = 0.90) compared to 81.1 % for total hippocampal volume (sensitivity = 75.2 %, specificity = 86.9 %, AUC = 0.897). Similar results for the combination of hippocampal subfields and total hippocampal volume were obtained when using the ADNI cohort as the test set and the AddNeuroMed cohort as the training set (Table 5). For further validation, we compared if subjects were classified differently between the different models for the combination of hippocampal subfields (for example classified as AD in one model and HC in another model). We compared the single cohort cross validated models with the combined cohort model and the single cohort models using the train/test approach. The results demonstrate that classification agreement for the different comparisons were high, lying between 89.5–98.8 % (Table 8).

**Table 8:** Comparison of subject classification between cohort models

	ANM and ANM/ADNI	ADNI and ANM/ADNI	ANM and ANMonADNI	ADNI on ADNIonANM
Total n	212	410	212	410
Same classification (n)	197	405	196	367
Same classification (%)	92.9	98.8	92.5	89.5
Different classification (n)	15	5	16	43
Different classification (%)	7.1	1.2	7.5	10.5

*ANM* AddNeuroMed cohort model, *ANM/ADNI* combined AddNeuroMed and ADNI cohort model, *ANMonADNI* AddNeuroMed cohort test set and ADNI training set, *ADNIonANM* ADNI cohort test set and AddNeuroMed cohort training set, *total n* AD and HC subjects, *Same classification* number of subjects predicted alike,  *% same classification* percentage of subjects predicted alike, *Different classification* number of subjects predicted differently, *Different classification (%)* percentage of subjects predicted differently

### Predicting MCI Conversion

Models previously constructed using AD and HC subjects from the combined cohort were applied to our large external test set of MCI subjects (*n* = 447) to predict future conversion to AD. These classifiers subsequently identified MCI subjects with an AD like brain structure (percentage classified as AD-like) or a healthy control-like brain structure (percentage classified as HC-like). During the 12 month follow up interval, 90 MCI subjects from the AddNeuroMed and ADNI cohorts met the clinical criteria for AD, and 357 MCI subjects remained clinically stable.

The combined subfield volumes classifier correctly identified 81.1 % of MCI converters from baseline images, with the presubiculum also correctly identifying 81.1 % of MCI-c with an AD-like pattern of subfield atrophy. In comparison, total hippocampal volume identified 76.7 % of MCI-c correctly. The predictive accuracies from the classifiers ranged between 56.7–81.1 % for MCI-c predictions (Table 9).

**Table 9:** MCI predictions using the baseline OPLS AD versus HC classifiers

	MCI-c classification (*n* = 90)^b^		MCI-s classification (*n* = 357)	
	AD like (%)*	HC like (%) **	AD like (%)*	HC like (%) **
CA1	**65.6 (59)**	34.4 (31)	48.2 (172)	**51.8 (185)**
CA2-3	**75.6 (68)**	24.4 (22)	52.1 (186)	**47.9 (171)**
CA4-DG	**74.4 (67)**	25.6 (23)	51.8 (185)	**48.2 (172)**
Fimbria	**56.7 (51)**	43.3 (39)	53.2 (190)	**46.8 (167)**
Presubiculum	**81.1 (73)**	18.9 (17)	51.0 (182)	**49.0 (175)**
Subiculum	**77.8 (70)**	22.2 (20)	51.3 (183)	**48.7 (174)**
Combined^a^	**81.1 (73)**	18.9 (17)	51.3 (183)	**48.7 (174)**
Total Hippocampal volume	**76.7 (69)**	23.3 (21)	49.9 (178)	**50.1 (179)**

*AD* Alzheimer’s disease, *MCI* mild cognitive impairment, *MCIc* MCI converter, *MCI-s* MCI stable, *HC* healthy control

* Sensitivity at each time point is the percentage of MCIc subjects correctly classified as AD in bold

** Specificity at each time point is the percentage of MCI-s subjects correctly classified as HC in bold

^a^The combined model used the combination of subfields for classification

^b^Only includes subjects that underwent conversion from MCI to AD at 12 months follow up

**Fig. 4:**
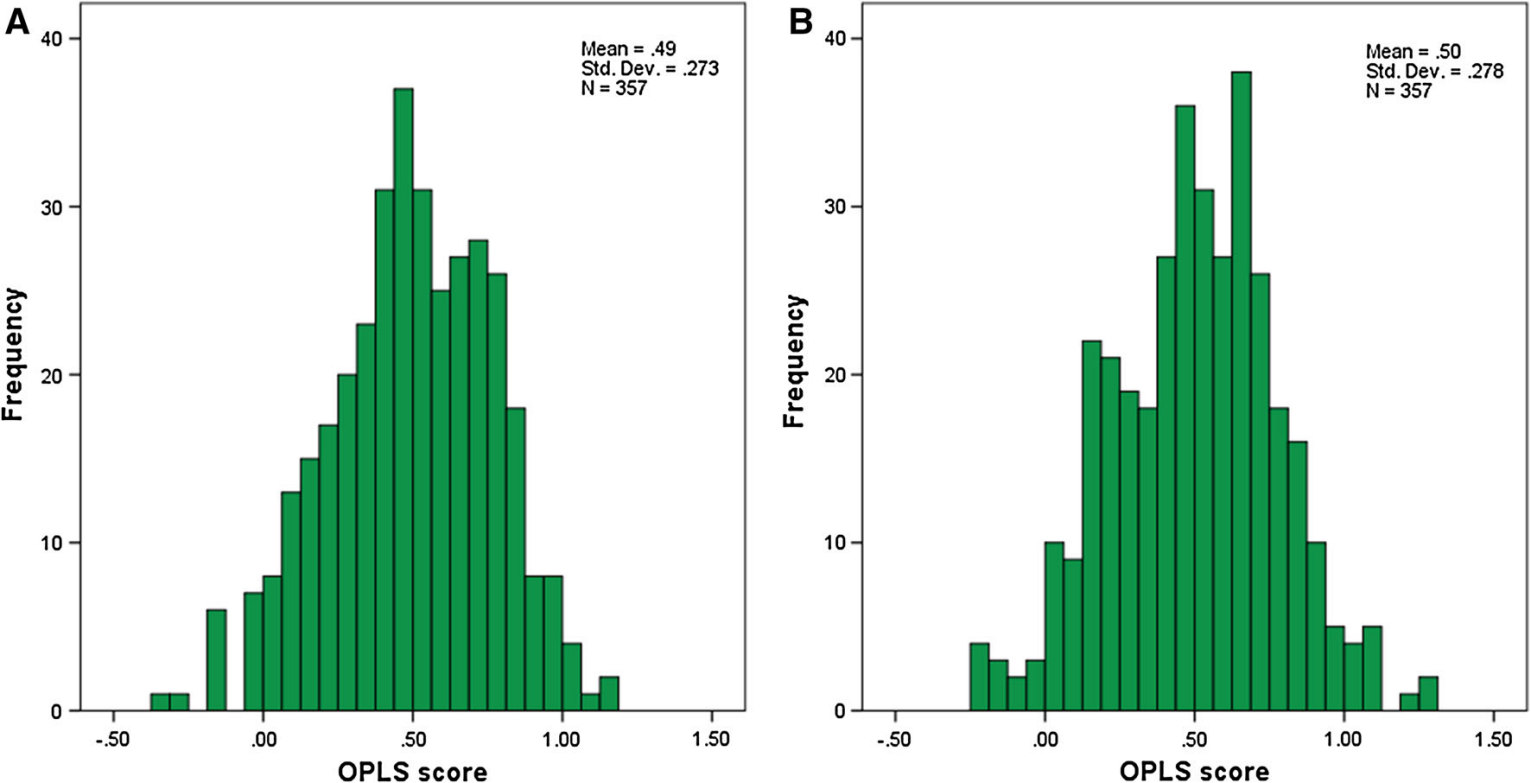
**a** OPLS scores from the total hippocampal volume classifier for MCI-s predictions, **b** OPLS scores from the combined subfields volume classifier for MCI-s predictions

However, a considerable number of MCI-s subjects were also predicted with an AD-like pattern of atrophy despite their clinically stable condition at 12 months follow up. For instance, the combined subfield volumes classifier, which was the most robust for MCI-c prediction, identified only 48.7 % of MCI-s with a HC-like subfield structure. A similar result was observed for the total hippocampal volume classifier which only identified 50.1 % of MCI-s correctly. Mean OPLS scores from the combined subfield volumes classifier and total hippocampal volume classifier were 0.50 ± 028 and 0.49 ± 0.27 (mean ± standard deviation) respectively (Fig. 4). As a result, differences in OPLS scores between the two classifiers were not statistically significant (Wilcoxon signed rank sum test, Z = −0.725, p = 0.469) despite the difference in AD-like MCI-c predictions. HC-like predictive accuracies in MCI-s predictions only ranged between 46.8–51.8 % which is because many of these MCI-s subjects will convert to AD at a future stage and already demonstrate an Alzheimer like pattern of hippocampal subfield atrophy.

## Discussion

Using an automated image analysis pipeline to explore the subfields of the hippocampus, we found that AD and MCI converters displayed a widespread pattern of subfield atrophy, including the bilateral subiculum, presubiculum and CA1 which have been reported in previous studies. Using the same image analysis approach, Hanseeuw et al. (2011) previously reported significant volume losses in the subiculum and CA2-3 region of the hippocampus in a small group of 15 amnestic MCI subjects and 15 healthy controls. We have extended this preliminary work to data from two large studies which together contain a more heterogeneous group of AD and MCI subjects that more accurately reflect the population of MCI and AD. The pattern of hippocampal volume loss that was found was wider than previous reports which have used either manual delineation techniques hippocampal subfield segmentation (Mueller and Weiner 2009; Mueller et al. 2010), 3D surface mapping (Apostolova et al. 2010) or shape analysis techniques (Csernansky et al. 2005; Costafreda et al. 2011) A similar pattern of subfield atrophy was observed for AD subjects and MCI converters suggesting that MCI converters may represent an imaging profile more similar to AD subjects than stable MCI. The pattern of hippocampal subfield loss, though wider than previously reported is in agreement with previous neuropathological studies reporting early neuronal loss in the subiculum, CA4-DG, and CA1 (West et al. 2004). Larger datasets are more likely to contain subjects with different types of atrophy which could explain the widespread pattern of subfield volume losses reported in the present study.

### Relationship Between Neuropsychological Test Scores and Hippocampal Subfields

A significant positive effect for both MMSE and ADAS-1 was found in relation to all hippocampal subfield volumes, indicating that subjects with lower MMSE scores and higher ADAS-1 scores had lower subfield volumes. This confirms the relationship between diffuse hippocampal volume loss and poorer neuropsychological test scores (Scheltens et al. 1992; Liu et al. 2009).

### Relationship Between Age, Gender, Education, *APOE* ε4 Genotype, and Hippocampal Subfields

Previous studies investigating the influence of age on hippocampal subfields have found significant negative effects associated with CA1 and CA2-3 subfield volumes (Mueller and Weiner 2009). Consistent with this previous work, using a larger dataset we also found a significant negative effect of age but in relation to all subfield volumes. However, years of education was only significantly associated with the right CA1 and right fimbria. Gender specific differences in the pattern of subfield volume loss were found, with female subjects demonstrating lower bilateral CA2-3, CA4-DG, fimbria, presubiculum and subiculum volumes. Previous work with AD patients suggests that gender specific differences in the rate of hippocampal volume loss are not entirely clear. For example, a previous study has reported a higher prevalence and incidence of AD in females (Barnes et al. 2005), whereas sex hormone differences have been suggested as an explanation of any gender divergence (Gouras et al. 2000). On the other hand, our findings suggest that carriers of the ε4 allele had smaller subfield volumes. which is in agreement with previous studies that have demonstrated a strong neuroanatomic effect of *APOE* ε4 genotype on the entire hippocampal region. (Jack et al. 1998; Reiman et al. 1998).

### AD and HC Classification

In this study we used the multivariate OPLS technique to distinguish between AD and control subjects. This method has previously been used for distinguishing between AD and control subjects (Westman et al. 2011a, b, c, d), as well as predicting conversion from MCI to AD using MRI regional measures and a combination of MRI regional measures and magnetic resonance spectroscopy (MRS) measures (Westman et al. 2010, 2011a, b, c, d). Hippocampal subfields have not been studied using this approach but several other studies have used alternative multivariate techniques including support vector machines, principal components analysis, and partial least squares to latent structures and linear discriminant analysis to analyse multiple MRI regional measures (Fan et al. 2008; McEvoy et al. 2009; Klöppel et al. 2008; Vemuri et al. 2008; Plant et al. 2010).

Studies that have attempted to distinguish between AD and control subjects have often done so using medial temporal structures such as the hippocampus and entorhinal cortex and reported accuracies of 80–90 % (Fox et al. 1996; Jack et al. 1992). Although prior studies have reported accuracies of up to 100 % in discriminating between AD and control subjects(Fan et al. 2008; Lerch et al. 2008), some studies used smaller samples, included more severely impaired AD patients or failed to cross-validate their findings. Here, using two large multicentre studies we segmented the hippocampus into its different subfields to examine whether subfield volumes could improve the sensitivity of MRI in detecting AD. The results suggest that the OPLS technique with fully automated hippocampal subfield volumes performs as accurately as total hippocampal volume, presubiculum volume and subiculum volume in distinguishing between AD and control subjects. The OPLS method which combined hippocampal subfield measures produced a classification accuracy of 81.7 % (sensitivity = 80.4 %, specificity = 82.8 %, AUC = 0.895), while total automated hippocampal volume produced an accuracy of 80.7 % (sensitivity 79.2 %, specificity 82.8 %, AUC = 0.887). Although significantly different, the magnitude of the difference is small and does not offer a particular advantage over hippocampal volume. Recent work has also found that the visual rating assessment of the medial temporal lobe produces accuracies that are comparable to that of manual hippocampal volume in distinguishing between AD and controls (Westman et al. 2011d).

Although our study is the first to use multivariate analysis of automated hippocampal subfields, previous research has examined the combination of automated regional cortical thicknesses and regional volumes in distinguishing between AD and control subjects using support vector machines and linear discriminant analysis (Vemuri et al. 2008; McEvoy et al. 2009).

### Predicting MCI Conversion

Building robust classification models on new and unseen data is of great importance for accurately predicting future MCI conversion to AD. MCI predictions were performed using AD vs. HC models in the combined ADNI and AddNeuroMed cohorts as classifiers and MCI subjects as our external validation test set. This approach has been applied previously and demonstrates how larger training sets can be used to assess MCI predictions that are more balanced in terms of sensitivity and specificity (Westman et al. 2011a). Previous studies in the neuroimaging literature utilising advanced methods of high dimensional pattern classification (Fan et al. 2008; Misra et al. 2009), and whole brain structural MRI (Karas et al. 2008; Davatzikos et al. 2010) have demonstrated the complexity of differential atrophy patterns observed in MCI-c and MCI-s subjects. Moreover, studies including our own have also shown heterogeneous patterns of brain atrophy exist in MCI subjects that convert to AD and those who remain clinically stable (Westman et al. 2011c; McEvoy et al. 2009). Consequently, hippocampal subfields were of interest following a small pilot study in MCI subjects (Hanseeuw et al. 2011).

Using a large external validation test set (*n* = 447) we sought to identify MCI subjects based on the similarity of their hippocampal subfield pattern to AD patients (% AD-like) or healthy control subjects (% HC-like). Unlike some previous studies, the large number of MCI subjects in our study served to more accurately represent the heterogeneity of MCI subjects and included both amnestic and non-amnestic subtypes.

The results demonstrated that the combination of subfield volumes and the presubiculum were the most robust classifiers, identifying 81.1 % of MCI-c correctly, and were better than using total hippocampal volume alone. However, a considerable number of MCI-s subjects were also predicted with AD-like patterns of atrophy despite having a clinically stable MCI condition at 12 months follow up. Although beyond the scope of the current study we intend in future to study longitudinal change in hippocampal subfield measures over longer follow up times in the ADNI cohort. The utility of structural MRI plays a key role in this domain and represents one of the 3 main biomarkers for AD diagnosis (Dubois et al. 2007; Frisoni et al. 2010). However, more focus needs to be addressed towards the standardisation of acquisition and analysis methods in order to facilitate the integration of findings across studies. Recently there has been much interest in exploring the combination of different MRI imaging techniques (i.e. Tensor based morphometry, cortical thicknesses and volumes) with cerebrospinal fluid (CSF) biomarkers, 18F-fluorodeoxyglucose PET, and clinical examination for classifying AD and predicting MCI conversion to AD (Wolz et al. 2011; Vemuri et al. 2009; Zhang et al. 2011; Furney et al. 2011). In regards to our study, a longer follow up time would be helpful to refine our estimates of model specificity for MCI-s predictions. A more robust algorithm that could potentially predict future MCI time to AD conversion would be of future interest to validate our findings in this present study.

## Conclusion

Hippocampal subfield volume loss in AD is widespread affecting regions such as the CA-1, subiculum, and presubiculum. Using an automated hippocampal subfield measurement technique we found prominent subfield volume losses in MCI converters and AD. Each of the subfield measures was related to both clinical predictors of AD (Age, gender, years of education, APOE E4 genotype) and cognitive scores (MMSE and ADAS-1 tests). Combined subfield volumes using the OPLS technique produced a similar classification accuracy to total hippocampal volume, presubiculum volume and subiculum volume in distinguishing between AD and HC subjects, but were more accurate than total hippocampal volume measurements at predicting MCI conversion to AD at 12 months.

## References

[CR1] Apostolova LG (2006). Conversion of mild cognitive impairment to Alzheimer disease predicted by hippocampal atrophy maps. Arch Neurol.

[CR2] Apostolova LG (2010). 3D comparison of low, intermediate, and advanced hippocampal atrophy in MCI. Hum Brain Mapp.

[CR3] Barnes LL (2005). Sex differences in the clinical manifestations of Alzheimer disease pathology. Arch Gen Psychiatry.

[CR4] Braak H, Braak E (1991). Neuropathological stageing of Alzheimer-related changes. Acta Neuropathol.

[CR5] Bylesjo M (2006). OPLS discriminant analysis : combining the strengths of PLS-DA and SIMCA classification y. J Chemom.

[CR6] Costafreda SG (2011). Automated hippocampal shape analysis predicts the onset of dementia in mild cognitive impairment. Neuroimage.

[CR7] Csernansky JG (2005). Preclinical detection of Alzheimer’s disease: hippocampal shape and volume predict dementia onset in the elderly. Neuroimage.

[CR8] Dale AM, Fischl B, Sereno MI (1999). Cortical surface-based analysis. I. Segmentation and surface reconstruction. Neuroimage.

[CR9] Davatzikos C (2010). Prediction of MCI to AD conversion, via MRI, CSF biomarkers, and pattern classification. Neurobiol Aging.

[CR10] Desikan RS (2009). Automated MRI measures identify individuals with mild cognitive impairment and Alzheimer’s disease. Brain.

[CR11] Devanand DP (2007). Hippocampal and entorhinal atrophy in mild cognitive impairment: prediction of Alzheimer disease. Neurology.

[CR12] Dubois B (2007). Research criteria for the diagnosis of Alzheimer’s disease: revising the NINCDS-ADRDA criteria. Lancet Neurol.

[CR13] Fan Y (2008). Spatial patterns of brain atrophy in MCI patients, identified via high-dimensional pattern classification, predict subsequent cognitive decline. Neuroimage.

[CR14] Fischl B (2002). Whole brain segmentation: automated labeling of neuroanatomical structures in the human brain. Neuron.

[CR15] Fischl B (2004). Sequence-independent segmentation of magnetic resonance images. Neuroimage.

[CR16] Fox NC (1996). Presymptomatic hippocampal atrophy in Alzheimer’s disease. A longitudinal MRI study. Brain.

[CR17] Frisoni GB (2010). The clinical use of structural MRI in Alzheimer disease. Nat Rev Neurol.

[CR18] Furney SJ (2011). Combinatorial markers of mild cognitive impairment conversion to Alzheimer’s disease–cytokines and MRI measures together predict disease progression. J Alzheimers Dis.

[CR19] Gouras GK (2000). Testosterone reduces neuronal secretion of Alzheimer’s β-amyloid peptides. Proc Natl Acad Sci USA.

[CR20] Hanseeuw BJ (2011). Mild cognitive impairment: differential atrophy in the hippocampal subfields. AJNR Am J Neuroradiol.

[CR21] Jack CR (1992). MR-based hippocampal volumetry in the diagnosis of Alzheimer’s disease. Neurology.

[CR22] Jack CR (1998). Hippocampal atrophy and apolipoprotein E genotype are independently associated with Alzheimer’s disease. Ann Neurol.

[CR23] Jack CR (2008). The Alzheimer’s disease neuroimaging initiative (ADNI): MRI methods. J Magn Reson Imaging.

[CR24] Karas G (2008). Amnestic mild cognitive impairment: structural MR imaging findings predictive of conversion to Alzheimer disease. AJNR Am J Neuroradiol.

[CR25] Klöppel S (2008). Automatic classification of MR scans in Alzheimer’s disease. Brain.

[CR26] Lerch JP (2008). Automated cortical thickness measurements from MRI can accurately separate Alzheimer’s patients from normal elderly controls. Neurobiol Aging.

[CR28] Liu Y (2009). Combination analysis of neuropsychological tests and structural MRI measures in differentiating AD, MCI and control groups-The AddNeuroMed study. Neurobiol Aging.

[CR29] Lovestone S (2009). AddNeuroMed–the European collaboration for the discovery of novel biomarkers for Alzheimer’s disease. Ann N Y Acad Sci.

[CR30] Mangialasche F (2010). High plasma levels of vitamin E forms and reduced Alzheimer’s disease risk in advanced age. J Alzheimers Dis.

[CR31] McEvoy LK (2009). Alzheimer disease: quantitative structural neuroimaging for detection and prediction of clinical and structural changes in mild cognitive impairment. Radiology.

[CR32] Misra C, Fan Y, Davatzikos C (2009). Baseline and longitudinal patterns of brain atrophy in MCI patients, and their use in prediction of short-term conversion to AD: results from ADNI. Neuroimage.

[CR33] Mueller SG, Weiner MW (2009). Selective effect of age, Apo e4, and Alzheimer’s disease on hippocampal subfields. Hippocampus.

[CR34] Mueller SG (2010). Hippocampal atrophy patterns in mild cognitive impairment and Alzheimer’s disease. Hum Brain Mapp.

[CR35] Plant C (2010). Automated detection of brain atrophy patterns based on MRI for the prediction of Alzheimer’s disease. Neuroimage.

[CR38] Reiman EM (1998). Hippocampal volumes in cognitively normal persons at genetic risk for Alzheimer’s disease. Ann Neurol.

[CR39] Robin X (2011). pROC: an open-source package for R and S + to analyze and compare ROC curves. Bioinformatics.

[CR40] Scheltens P (1992). Atrophy of medial temporal lobes on MRI in “probable” Alzheimer’s disease and normal ageing: diagnostic value and neuropsychological correlates. J Neurol Neurosurg Psychiatry.

[CR41] Ségonne F (2004). A hybrid approach to the skull stripping problem in MRI. Neuroimage.

[CR42] Simmons A (2009). MRI measures of Alzheimer’s disease and the AddNeuroMed study. Ann N Y Acad Sci.

[CR43] Simmons A (2011). The AddNeuroMed framework for multi-centre MRI assessment of Alzheimer’s disease: experience from the first 24 months. Int J Geriatr Psychiatry.

[CR44] Spulber G (2013). An MRI-based index to measure the severity of Alzheimer’s disease-like structural pattern in subjects with mild cognitive impairment. J Intern Med.

[CR45] Trygg J, Wold S (2002). Orthogonal projections to latent structures (O-PLS). J Chemom.

[CR46] Van Leemput K (2009). Automated segmentation of hippocampal subfields from ultra-high resolution in vivo MRI. Hippocampus.

[CR47] Vemuri P (2008). Alzheimer’s disease diagnosis in individual subjects using structural MR images: validation studies. Neuroimage.

[CR48] Vemuri P (2009). MRI and CSF biomarkers in normal, MCI, and AD subjects: predicting future clinical change. Neurology.

[CR49] West MJ (2004). Hippocampal neurons in pre-clinical Alzheimer’s disease. Neurobiol Aging.

[CR100] Westman E, Wahlund LO, Foy C et al (2010) Combining MRI and MRS to distinguish between Alzheimer’s disease and healthy controls. J Alzheimers Dis 22(1):171–181 10.3233/JAD-2010-100168 20847449

[CR50] Westman E, Simmons A, Muehlboeck J-S (2011). AddNeuroMed and ADNI: similar patterns of Alzheimer’s atrophy and automated MRI classification accuracy in Europe and North America. Neuroimage.

[CR51] Westman E, Wahlund L, Foy C (2011). Magnetic Resonance Imaging and Magnetic Resonance Spectroscopy for Detection of Early Alzheimer’ s Disease. J Alzheimers Dis.

[CR52] Westman E, Simmons A, Zhang Y (2011). Multivariate analysis of MRI data for Alzheimer’s disease, mild cognitive impairment and healthy controls. Neuroimage.

[CR53] Westman E, Cavallin L, Muehlboeck J-S (2011). Sensitivity and Specificity of Medial Temporal Lobe Visual Ratings and Multivariate Regional MRI Classification in Alzheimer’s Disease J. Laks, ed. PLoS One.

[CR54] Westman E, Muehlboeck J-S, Simmons A (2012). Combining MRI and CSF measures for classification of Alzheimer’s disease and prediction of mild cognitive impairment conversion. Neuroimage.

[CR55] Westman E (2013). Regional magnetic resonance imaging measures for multivariate analysis in Alzheimer’s disease and mild cognitive impairment. Brain Topogr.

[CR56] Wiklund S (2008). Visualization of GC/TOF-MS-based metabolomics data for identification of biochemically interesting compounds using OPLS class models. Anal Chem.

[CR57] Wolz R (2011). Multi-method analysis of MRI images in early diagnostics of Alzheimer’s disease. PLoS One.

[CR58] Zhang D (2011). Multimodal classification of Alzheimer’s disease and mild cognitive impairment. Neuroimage.

